# Comparison of pediatric radiation dose and vessel visibility on angiographic systems using piglets as a surrogate: antiscatter grid removal vs. lower detector air kerma settings with a grid — a preclinical investigation

**DOI:** 10.1120/jacmp.v16i5.5379

**Published:** 2015-09-08

**Authors:** Keith J. Strauss, John M. Racadio, Todd A. Abruzzo, Neil D. Johnson, Manish N. Patel, Kamlesh U. Kukreja, Mark. J. H. den Hartog, Bart P.A. Hoornaert, Rami A. Nachabe

**Affiliations:** ^1^ Department of Radiology Cincinnati Children's Hospital Medical Center, University of Cincinnati School of Medicine Cincinnati OH; ^2^ Department of Radiology Texas Children's Hospital, Baylor College of Medicine Houston TX; ^3^ Interventional X‐ray Department Philips Healthcare Best The Netherlands

**Keywords:** antiscatter grid, radiation dose, image quality, pediatric

## Abstract

The purpose of this study was to reduce pediatric doses while maintaining or improving image quality scores without removing the grid from X‐ray beam. This study was approved by the Institutional Animal Care and Use Committee. Three piglets (5, 14, and 20 kg) were imaged using six different selectable detector air kerma ($K_{\text{air}}$) per frame values (100%, 70%, 50%, 35%, 25%, 17.5%) with and without the grid. Number of distal branches visualized with diagnostic confidence relative to the injected vessel defined image quality score. Five pediatric interventional radiologists evaluated all images. Image quality score and piglet $K_{\text{air}}$ were statistically compared using analysis of variance and receiver operating curve analysis to define the preferred dose setting and use of grid for a visibility of 2nd and 3rd order vessel branches. Grid removal reduced both dose to subject and imaging quality by 26%. Third order branches could only be visualized with the grid present; 100% detector $K_{\text{air}}$ was required for smallest pig, while 70% detector $K_{\text{air}}$ was adequate for the two larger pigs. Second order branches could be visualized with grid at 17.5% detector $K_{\text{air}}$ for all three pig sizes. Without the grid, 50%, 35%, and 35% detector $K_{\text{air}}$ were required for smallest to largest pig, respectively. Grid removal reduces both dose and image quality score. Image quality scores can be maintained with less dose to subject with the grid in the beam as opposed to removed. Smaller anatomy requires more dose to the detector to achieve the same image quality score.

PACS numbers: 87.53.Bn, 87.57.N‐, 87.57.cj, 87.59.cf, 87.59.Dj

## I. INTRODUCTION

One of the cornerstones of the *Image Gently* campaign is the need to carefully manage both the radiation dose and image quality of any pediatric imaging procedure that uses ionizing radiation.^1^, ^2^, ^3^ To this end, numerous authors over a number of years have recommended removing the antiscatter grid from the X‐ray beam during fluoroscopic examinations of small children.^4^, ^5^, ^6^, ^7^, ^8^, ^9^, ^10^, ^11^, ^12^, ^13^, ^14^, ^15^, ^16^, ^17^ While some authors have quantified the reduction in patient dose to these pediatric patients,^7^, ^13^, ^16^, ^17^, ^18^, ^19^ the loss of image quality associated with this reduction in patient dose has been measured only on a limited basis^7^, ^9^, ^14^, ^16^, ^17^, ^20^ with phantoms as opposed to live models. In addition, the studies that do exist^7^, ^20^, ^21^ tend to address the pediatric interventional cardiology laboratory application as opposed to the pediatric interventional radiology laboratory.

Removal of the grid in interventional radiology or cardiology laboratory to reduce pediatric patient dose may not be ideal or possible. The reduction in pediatric patient dose is achieved at the expense of image quality due to increased scatter at the detector. Removal of the grid is impossible on some angiographic systems without the use of tools. Removing the grid on some of these units subjects the fragile input surface of the image intensifier, now exposed, to unacceptable risk of damage from collisions.

We hypothesized that state‐of‐the‐art image processing technology capable of higher image quality scores at lower piglet doses might demonstrate the elimination of the need to remove the grid during angiographic studies of small patients. The purpose of this investigation was to reduce the patient dose, while maintaining or improving pediatric image quality scores, without removal of the grid from the X‐ray beam during creation of DSA recorded images (fluorography) during interventional procedures of pediatric patients.

## II. MATERIALS AND METHODS

### A. Animal study

All procedures described in the study were performed after approval of the local Institutional Animal Care and Use Committee. Three different sized piglets of 5, 14, and 19.5 kg were used. The piglets' measured body thicknesses of 9, 14, and 17 cm, respectively, correspond to a small newborn, average three‐year‐old, and average five‐year‐old human abdomen.^(22)^ The animals were sedated with intramuscular injections of xylazine (2 mg/kg, Fort Dodge Animal Health, Fort Dodge, IA), ketamine (20 mg/kg, Fort Dodge Animal Health), and buprenorphine (0.02 mg/kg, Reckitt Benckiser Pharmaceuticals Inc., Richmond, VA). General anesthesia with 2% isoflurane was applied to the animals. The piglets were euthanized with pentobarbital sodium (1 ml/10 lbs intravenous injection (Fatal‐Plus, Vortech Pharmaceuticals, Dearborn, MI) at the conclusion of the imaging session.

### B. Image acquisition

Digital subtraction angiography (DSA) was performed with a power injector using iodinated contrast (Optiray 350, Covidien, Hazelwood, MO) with the catheter tip at four different anatomic locations: the abdominal aorta and the origins of the renal, common hepatic, and superior mesenteric arteries. The DSA runs were performed using an acquisition protocol of two frames per second (fps) for 3 s, followed by one fps for 6 secs, and 0.5 fps until the DSA run was stopped. The angiographic system that was used is a flat‐panel detector C‐arm (Philips AlluraClarity, Best, The Netherlands) with ClarityIQ imaging technology.^23^, ^24^ Both the features of the image processing engine and acquisition chain parameters (beam filtering, pulse width, pulse rate, focal spot size selection, and required detector dose) of the fluoroscope were leveraged by the study design to manage both image quality and piglet dose during the study.

DSA acquisitions were performed with and without the grid provided by the vendor (12:1 grid ratio; 105 cm focal distance; 44 lines per cm) in place at six different air kerma, $K_{\text{air}}$, settings at the entrance plane of the detector (detector $K_{\text{air}}$ setting). The detector $K_{\text{air}}$ was modified by changing the product of tube current and exposure time (mAs). The recommended detector $K_{\text{air}}$, 1.0 μGy was chosen as 100% for the largest field of view (FoV) of the image receptor. Other settings available for the largest FoV included 70%, 50%, 35%, 25%, and 17.5%. The FoV and the collimation of the X‐ray beam were constant for each anatomical location in a given sized piglet. While the beam area was fixed for each of the six dose settings for a given anatomy and piglet size, FoV for the smallest piglet was typically 1.5 times smaller than for the medium and large piglets^(24)^ to allow proper visualization of the smaller vessels, which increased the detector $K_{\text{air}}$ for the smallest piglet by a factor of 1.5. The X‐ray tube voltage was held constant at 73 kV with 0.4 mm of copper and 1.0 mm of aluminum inserted in the beam. A small focal spot of 0.4 mm nominal size was used. The fixed high voltage and added filter throughout the study ensured that, for a given piglet size and grid configuration, the percent change of $K_{\text{air}}$ at the entrance plane of a piglet is equal to the percent change of detector $K_{\text{air}}$. The automatic brightness control system of the fluoroscope was allowed to adjust the product of tube current and pulse width used for each DSA image to deliver the specified detector $K_{\text{air}}$ as the piglet size and grid configuration changed. A total of 144 DSA runs along with their radiation dose structured reports (RDSR) were stored on a Picture Archiving and Communication System (PACS).

The $K_{\text{air}}$ at the interventional reference point (IRP) was taken from the RDSR. By definition, the displayed values can contain up to ±35% error. The local institutional medical physicist (one of the authors) measured the $K_{\text{air}}$ at the IRP to obtain a more accurate estimate. The error of the calibrated value is determined by the propagation of all the errors of the measurement. The calibration of the dosimeter used was ±5%. There are additional errors in placing the ionization chamber at the IRP. However, if the measurements are carefully performed, the total error of the calibration should not exceed ±10%. The ratio of the $K_{\text{air}}$ measured to the $K_{\text{air}}$ reported in the RDSR was found to be 1.03. Since the vertical position of the tabletop was unchanged for the three piglets, careful measurements were made at the beginning of the study to determine that the tabletop (entrance plane of each piglet) was less than 1 cm from the isocenter towards the focal spot. This value was averaged by the number of frames during fluorography to estimate the average entrance air kerma per frame to each piglet. This index of patient dose is called “piglet $K_{\text{air}}/\text{fr}$” throughout this paper.

### C. Image scoring

A five‐point ordinal scale published elsewhere^(24)^ was used as a metric for image quality scoring based on the order of vessel branches relative to the injected vessel that are clearly visible. A score of 0 was assigned to nondiagnostic runs. Scores of 1, 2, and 3 corresponded to complete diagnostic confidence in evaluation of 1st, 2nd, and 3rd order branches relative to the injected vessel, respectively. A score of 4 was assigned to images that could depict more than 3rd order branches. Five pediatric interventional radiologists, with 6, 9, 13, 15, and 22 years of angiographic interpretive experience, scored the runs displayed on a diagnostic imaging monitor. All information pertaining to the radiographic technique used for the images was removed from the displayed images. Each physician scored each image twice separated by a minimum of one week to avoid memory bias. The physicians had complete freedom to manipulate the images (e.g., contrast, brightness, magnification, speed of display including pausing) as they would do clinically.

### D. Statistical analysis

For each DSA run, the average of the scores from all five reviewers was considered for statistical analysis. The intracorrelation coefficient and the corresponding 95% confidence interval (CI) were computed to assess interrater agreement of image quality scoring.

Linear regression along with 95% confidence bounds were computed to evaluate the piglet $K_{\text{air}}/\text{fr}$ reduction that was achieved when removing the grid at all six dose settings. A similar analysis was applied to the image quality scores to evaluate change in image quality with removal of grid. The pairwise Wilcoxon test was applied to evaluate statistical differences in pediatric dose index and image quality scores between presence and absence of the grid.

Receiver operating characteristic (ROC) analysis was used to investigate specific image quality requirements with the grid present or absent when the minimum required vessel visibility was set to 2 and to 3. ROC curves were computed for all animal sizes, as well as per different animal size. Area under the ROC curve (AUC) along with the standard errors (SE) were computed and the optimum mAs setting for a specific vessel visibility requirement was defined as the cutoff point on the ROC curve for the best sensitivity and specificity.^(25)^

Repeated two‐way analysis of variance, with dose setting and presence or absence of grid being the two main variables, was applied to pediatric dose index and image quality in order to evaluate the statistical differences. In the case of statistical difference, Tukey's *post hoc* test for multiple comparisons correction was applied. Statistical calculations were performed using MATLAB version 7.12 software (MathWorks, Natick, MA) and a p‐value smaller than 0.05 was considered statistically significant.

## III. RESULTS

The overall interrater agreement was 0.81 (CI: 0.60–0.90) indicating excellent agreement.^(26)^ The range of diameters of the injected vessels (aorta, superior mesenteric, common hepatic, and renal) and the 1st through 4th order branching vessel diameter ranges are summarized for each pig in Table 1

**Figure 1:**
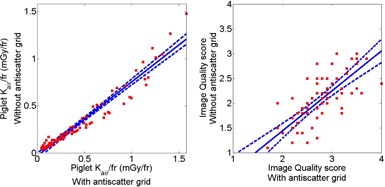
Comparison of piglet $K_{\text{air}}/\text{fr}$ at entrance plane of the piglet (left figure) and image quality score (right figure) when the grid is in place vs. when it is removed. The straight line corresponds to the linear regression, whereas the dashed lines delineate the 95% confidence bounds. The linear regression equations without grid vs. with grid for pediatric dose index (left) is y=0.78x−0.03 and for image quality score (right) is y=0.81x−0.17. Each plot contains 72 data points from the combinations of three piglets, four anatomical locations, and six detector AK levels.

Removal of the grid reduced mean piglet $K_{\text{air}}/\text{fr}$ by 26% (0.48±0.40 with grid vs. 0.35±0.32 mGy/fr without grid, p<0.05), while mean image quality scores decreased by 26% (2.7±0.5 with grid vs. 2.0±0.5 without grid, p<0.0001). Linear regression plots comparing piglet $K_{\text{air}}/\text{fr}$ and image quality scores with and without grid are shown in Fig. 1. Each of the two plots contains 72 data points from all the combinations of three piglets, four different anatomical locations within the piglets, and six different detector $K_{\text{air}}$ levels. Since the linear regression of each plot is <1, the plots illustrate increased piglet $K_{\text{air}}/\text{fr}$ and improved image quality scores when the grid is inserted in the X‐ray beam.

**Figure 2:**
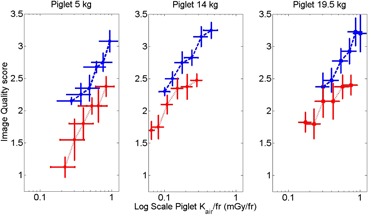
Image quality score vs. piglet $K_{\text{air}}/\text{fr}$ with and without the grid for the 5, 14, and 19.5 kg piglets, respectively. Each point represents a sequentially greater detector $K_{\text{air}}$ setting (17.5%, 25%, 35%, 50%, 70%, and 100%, from left to right).

**Figure 3:**
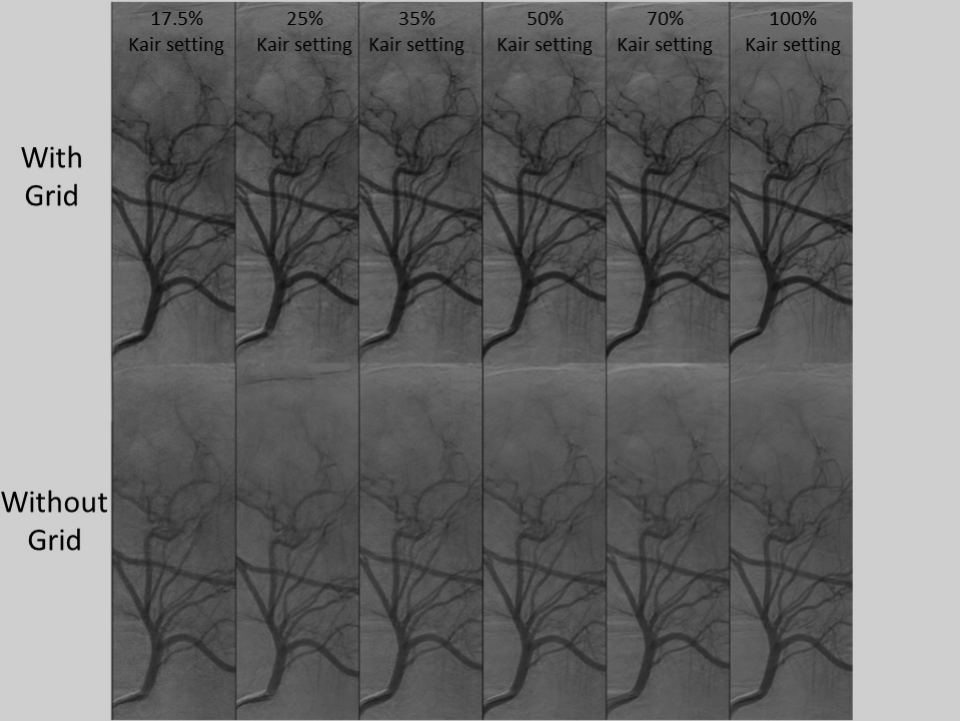
DSA runs with (top row) and without (bottom row) the grid in place. Left to right images correspond to acquisitions performed at detector $K_{\text{air}}$ settings of 17.5%, 25%, 35%, 50%, 70%, and 100%, respectively. Images shown are for 14 kg piglet.

The ROC analysis per piglet size demonstrated that the grid should not be removed regardless of the animal size when an image quality score of more than 3 is required (Table 2). The optimum detector $K_{\text{air}}$ setting is 100% for the smallest piglet and 70% for the medium and largest piglets. When a minimum of two vessels must be visible to make a reliable diagnosis, the lowest detector $K_{\text{air}}$ can be used with the grid present. With the grid absent, the optimum detector $K_{\text{air}}$ setting for the smallest piglet is 50% and 35% for the medium and largest piglets. The mean image quality score versus mean piglet $K_{\text{air}}/\text{fr}$ for each detector $K_{\text{air}}$ setting with and without the grid for each animal size is depicted in Fig. 2. As an example, representative images are provided in Fig. 3 that correspond to angiographic runs performed by injecting contrast into the common hepatic artery of the medium sized piglet (three‐year‐old human abdomen size).

**Table 1:** Diameter of injected vessels and branches in each piglet

	*Vessel Diameter (mm)*				
	*Injected Vessel*	*1st Order Branch*	*2nd Order Branch*	*3rd Order Branch*	*4th Order Branch*
Piglet 5 kg	2.2–6.0	1.6–2.4	0.5–1.6	0–0.6	0–0.2
Piglet 14 kg	3.0–8.3	1.8–3.7	0.7–2.0	0.2–1.0	0–0.4
Piglet 19.5 kg	3.9–8.9	2.5–4.7	1.4–2.5	0.5–1.5	0–0.8

Figure 2 depicts the mean ± SE of image quality scores versus mean piglet $K_{\text{air}}/\text{fr}$ ± SE for the three piglets at the four anatomical sites within each piglet and each detector $K_{\text{air}}$ setting when the grid was present and absent. Removing the grid at the 100% detector $K_{\text{air}}$ setting produced a piglet $K_{\text{air}}/\text{fr}$ reduction of 22% (0.80±0.14 vs. 0.62±0.12 mGy/fr). However, the image quality score was significantly degraded as well from 3.2±0.1 to 2.4±0.1 (p<0.05). The image quality score at the lowest detector $K_{\text{air}}$ setting (17.5%) with the grid present was equivalent to the image quality score at 70% detector $K_{\text{air}}$ setting without the grid (2.3±0.1 vs. 2.3±0.1). The image quality score without the grid is matched to the result with the grid with a significantly lower piglet $K_{\text{air}}/\text{fr}$ (0.23±0.05 vs. 0.48±0.10 mGy/fr, p<0.05) because the detector $K_{\text{air}}$ setting is significantly lower (17.5% vs. 70%). At the lowest detector $K_{\text{air}}$ setting, removing the grid significantly reduced the image quality score from 2.3±0.1 to 1.5±0.1 (p<0.05), but the reduction in piglet $K_{\text{air}}/\text{fr}$ was not statistically significant, 0.23±0.05 to 0.15±0.03 mGy/fr (p=0.06).

**Table 2:** ROC analysis for DSA runs of each subject size with an image quality score higher than 3 and higher than 2 with the grid in place or removed

			*AUC ± SE*	*Optimum Detector* $K_{\text{air}}$ *Setting*	*Piglet* $K_{\text{air}}/\text{fr}$ *± SE (mGy/fr)*	*Image Quality Score ± SE*
Piglet 5 kg	Image Quality ≥3	with grid	0.95±0.08	100%	0.94±0.26	3.1±0.2
		without grid	NA	None	NA	NA
	Image Quality ≥2	with grid	0.79±0.12	17.5%	0.27±0.10	2.2±0.1
	without grid		0.78±0.11	50%	0.53±0.19	2.1±0.1
Piglet 14 kg	Image Quality ≥3	with grid	0.80±0.12	70%	0.33±0.07	3.2±0.2
		without grid	NA	None	NA	NA
	Image Quality ≥2	with grid	1.00±0.00	17.5%	0.10±0.02	2.3±0.1
		without grid	0.81±0.09	35%	0.11±0.02	2.1±0.1
Piglet 19.5 kg	Image Quality ≥3	with grid	0.75±0.11	70%	0.87±0.19	3.2±0.2
		without grid	NA	None	NA	NA
	Image Quality ≥2	with grid	0.91±0.07	17.5%	0.30±0.06	2.4±0.2
		without grid	0.81±0.09	35%	0.31±0.07	2.2±0.2

AUC = area under curve; SE = standard error; NA = image quality score ‘not achievable’ without grid in place.

**Figure 4:**
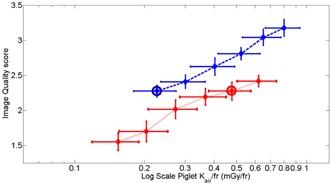
Image quality score vs. the logarithm of piglet $K_{\text{air}}/\text{fr}$. Each point represents the average of five readers from four anatomical sites from each of three piglets. Each point represents a sequentially greater piglet $K_{\text{air}}/\text{fr}$ which is proportional for a given piglet size to the detector $K_{\text{air}}$ setting (17.5%, 25%, 35%, 50%, 70%, and 100%, from left to right). Note that with the grid in place, the image quality score using the lowest detector $K_{\text{air}}/\text{fr}$ (17.5%, circled blue dot) is equivalent to the image quality score using a detector $K_{\text{air}}$ setting of 70% without the grid (circled red dot); both image quality scores are 2.3. However, the piglet $K_{\text{air}}/\text{fr}$ is significantly lower with the grid in place (0.23 vs. 0.48 mGy/fr) because of the lower detector $K_{\text{air}}$ setting used (17.5% vs. 70%). The log piglet $K_{\text{air}}/\text{fr}$ spreads out/compresses the data points on the left vs. the right of the plot, respectively, to coincide with small and large changes in the data points.

The results of ROC analysis, assuming a score of at least 2 or 3 as a minimum required threshold for complete diagnostic confidence, are summarized in Table 3. For an image quality score threshold of 3, the optimum detector $K_{\text{air}}$ setting is 70% (AUC=0.81±0.07); grid removal is not suitable for any of the detector $K_{\text{air}}$ settings (AUC<0.5). For an image quality score threshold of 2, the AUCs with and without grid are 0.83±0.07 and 0.78±0.05, respectively, yielding an optimum detector $K_{\text{air}}$ setting of 17.5% with grid and 50% without grid. While the image quality scores were similar (2.3 vs. 2.2) with and without the grid at these optimum detector $K_{\text{air}}$ settings, the piglet $K_{\text{air}}/\text{fr}$ was significantly lower with the grid (0.23±0.05 mGy/fr vs. 0.37±0.08 mGy/fr, p<0.05).

**Table 3:** ROC analysis of pooled data for DSA runs with an image quality score higher than 3 and higher than 2 with the grid in place or removed

		*AUC ± SE*	*Optimum Detector* $K_{\text{air}}$ *setting*	*Piglet* $K_{\text{air}}/\text{fr}$ *± SE (mGy/fr)*	*Image Quality Score ± SE*
Image Quality ≥3	with grid	0.81±0.07	70%	0.65±0.12	3.0±0.1
	without grid	NA	None	NA	NA
Image Quality ≥2	with grid	0.83±0.07	17.5%	0.23±0.05	2.3±0.1
	without grid	0.78±0.05	50%	0.37±0.08	2.2±0.1

AUC = area under curve; SE = standard error; NA = image quality score ‘not achievable’ without grid in place.

## IV. DISCUSSION

The typical diagnostic task of angiography is detection of a vessel containing iodinated contrast agent. The amount of contrast agent and resultant subject contrast in a specific vessel is a function of the product of the concentration of the contrast and the diameter of the vessel. Hence the amount of radiographic contrast is larger for 1st and 2nd order vessels, but smaller for 3rd and 4th order vessels due to their smaller diameter. The imaging task is likely to be noise‐limited for smaller visualized low‐contrast vessels. This explains the need for increased detector $K_{\text{air}}$ settings without the grid for an image quality score >2 and with the grid for an image quality score >3, which increases the piglet $K_{\text{air}}/\text{fr}$, to achieve the same image quality score for the smallest piglet with the smallest diameter vessels (1, 2).

Since smaller volumes of patient tissue (mass) are irradiated in small pediatric as opposed to adult patients, production of scatter is reduced. Conventionally, pediatric imaging has been viewed as an opportunity to reduce patient dose by removing the antiscatter grid. The reduction of pediatric patient dose has been quantified numerous times,^7^, ^13^, ^16^, ^17^, ^18^, ^19^ but the loss of image quality, due to the increase in scatter (increased scatter/primary) when evaluated, was quantified with phantoms as opposed to live animal models.^7^, ^9^, ^14^, ^16^, ^17^, ^20^ This manuscript suggests the alternative of leaving the grid in place to preserve contrast, but lowering detector $K_{\text{air}}$ setting to reduce piglet $K_{\text{air}}/\text{fr}$ which reduces the piglet's dose. This produces images with both more contrast and noise, but advanced image processing reduces perceived noise while maintaining contrast to preserve detection of the smaller vessels.

While each of the piglets had a different thickness, it is not unreasonable to pool the data from the three pigs in Fig. 4 and Table 3. The results of the paired statistical tests applied to the data indicated that pooling the data results in a statistically fair comparison. The differences of AUC and image quality scores of the three different piglets, Table 2, were not statistically significant (p=0.2). The optimum detector $K_{\text{air}}$ setting is only increased by 1 step for the smallest piglet for grid:image quality score ≥3 and for nogrid:image quality score ≥2, which explains some of the increase in piglet $K_{\text{air}}$. However, the biggest contributor to the increase in piglet $K_{\text{air}}/\text{fr}$ for the smallest piglet is due to the decrease in FoV by a factor of 1.5, which increases the detector $K_{\text{air}}$ by approximately 50%. Provided the detector $K_{\text{air}}$ is unchanged, one is tempted to assume that the average dose is largest for the largest pig because it drives the automatic brightness control system to the largest radiation output. However, our largest piglet, 17 cm, attenuates four to five times more radiation (energy) than the smallest piglet, 9 cm, due to increased attenuation in the largest piglet, but this increase is largely cancelled out since the mass of our smallest piglet was 25% of the largest piglet and dose is defined as the energy/mass.

If visualization of 2nd order vessels is adequate to conduct a clinical examination, this study illustrates that an image quality score of 2.3 can be achieved with or without the grid in the X‐ray beam. The required piglet $K_{\text{air}}$ to achieve this image quality score with the grid is 48% of the required piglet $K_{\text{air}}$ without grid (0.23 mGy frame with grid and 0.48 mGy/fr without grid, Fig 4). In addition, this study demonstrated that diagnostically confident visualization of 3rd order vessels requires the presence of the grid for the range of piglet sizes that were examined.

These results suggest opportunities for the operator to reduce the piglet $K_{\text{air}}/\text{fr}$ without loss of the image quality score depending on the complexity of the imaging examination. There are pediatric interventional radiology applications for which diagnostically confident visualization of 2nd order vessels is adequate to complete the study. Three examples are a Wada test (known as intracarotid sodium amobarbital procedure) to confirm catheter tip location and flow dynamics within the internal carotid artery prior to amobarbitol injection; a varicocele and venous malformation mapping prior to sclerotherapy embolization; or intraprocedure control angiograms to assess interval progress in vessel occlusion during embolization procedures. For such procedures, the operator can use the grid and cut the detector $K_{\text{air}}$ setting in half compared to without the grid while maintaining similar image quality scores. For other studies for which diagnostically confident visualization of 3rd order vessels is required (e.g., renal arteriography for possible renal artery stenosis, as stenoses in children can be in 3rd order branches), visceral angiography for gastrointestinal bleeding, and initial diagnostic angiography of high‐flow vascular malformations and tumors prior to embolization procedures, a significant improvement in image quality score is required. Therefore, the piglet $K_{\text{air}}/\text{fr}$ must be increased 2.8 times (0.65 mGy/fr with grid and image quality ≥3 vs. 0.23 mGy/fr with grid and image quality ≥2, Table 2).

The clinical opportunities discussed in the previous paragraph require the estimation of the entrance skin $K_{\text{air}}/\text{fr}$, which is dependent on patient thickness, the location of the patient with respect to the focal spot and image receptor of the fluoroscope, the detector $K_{\text{air}}/\text{fr}$ setting during fluorography, and the specific examination. This requires creation of an extensive set of protocols and testing that account for these multiple variables prior to their application to clinical examinations.

The authors of this study are not aware of any data in the literature on this topic from an interventional radiology lab. Three different studies performed with phantoms simulating patients^17^, ^19^, ^20^ were performed in interventional cardiology labs; the authors of those studies recommended the removal of the grid for small patients. Ubeda et al.^(20)^ evaluated the difference in dose and image quality with and without the grid in place for four simulated patient thicknesses of 8, 12, 18, and 24 cm. They suggested that, for a patient thickness of 8 cm, the grid could potentially be removed without noticeable loss of image quality, but it should remain in place for patient thicknesses of 12 cm and above.

We believe that the drawbacks of phantoms prevent proper pediatric patient $K_{\text{air}}/\text{fr}$ and image quality score evaluation. First, homogeneous phantoms generate different scatter patterns than the nonhomogeneous organs of a patient. Second, phantoms do not contain the anatomical structures found in patients; the appearance of phantom images lacks the subtle characteristics clinicians expect to see and are comfortable rating in clinical images. Third, static phantoms fail to model clinical motion (e.g., respiration, bowel motion). Consequently, we believe an animal study provides a more accurate clinical model on which to evaluate pediatric patient $K_{\text{air}}/\text{fr}$ and image quality score for repeated measurements with and without the grid.

Our study analyzes the impact of the presence or absence of the grid on both image quality scores and piglet $K_{\text{air}}/\text{fr}$ only during fluorography. Our fluorography test method would not provide clinical information about the adequacy of fluoroscopic image quality since contrast is not injected into the vascular during fuoroscopy. Since piglet $K_{\text{air}}/\text{fr}$ during fluorography is reduced only 26% with grid removed, a reduction of detector $K_{\text{air}}$ setting during fluoroscopy of 26% with grid inserted should match the piglet $k_{\text{air}}/\text{fr}$ during fluoroscopy with 100% detector $K_{\text{air}}$ setting with grid removed. If grid removal prevents adequate visualization of interventional guide wires and other high‐contrast devices, radiologists should object; the radiologists know the appropriate appearance of these devices in a fluoroscopic image. Fluoroscopy is a different imaging task than identifying the presence or absence of subtle anatomical abnormalities from a fluorographic image. Therefore, a clinical trial that reduces the detector $K_{\text{air}}$ setting in small steps can occur. When the radiologists begin to object to the quality of the fluoroscopic images, add at least 1 step of detector $K_{\text{air}}$ setting back into the system to insure diagnostic fluoroscopy image quality. Finally, verify that the final detector $K_{\text{air}}$ setting does not exceed 74% of the original detector $K_{\text{air}}$ during fluoroscopy.

Our study contains several other limitations. Collimation was purposely controlled and held constant. The effect of collimation on piglet $K_{\text{air}}/\text{fr}$ and image quality score was not evaluated. This study was performed with AlluraClarity, an image processing system that improves image quality scores at reduced dose settings. Our results might extrapolate to other systems with less image processing capability that operate at higher patient dose levels; however, this needs to be confirmed by performing a similar study with these other systems.

## V. CONCLUSIONS

While removal of the grid from the X‐ray beam reduces the piglet's dose, this benefit is achieved at the expense of image quality score due to an increase of scatter radiation in the image. Smaller subjects require more entrance $K_{\text{air}}/\text{fr}$ during fluorography to achieve the same image quality scores of larger subjects. High image quality scores (confident diagnostic visualization of 3rd order vessels) can only be achieved with the grid in the X‐ray beam. Adequate image quality scores (confident diagnostic visualization of 2nd order vessels) can be achieved with or without the grid in the X‐ray beam. The required piglet $K_{\text{air}}/\text{fr}$ with the grid in the X‐ray beam is half the required piglet $K_{\text{air}}/\text{fr}$ without the grid to achieve the same image quality score. In the future, configurations of interventional fluoroscopic equipment that appropriately manage the detector $K_{\text{air}}$ setting for both fluoroscopy and fluorography relative to patient size and type of study may provide improved image quality at reduced patient doses with no need to remove the grid from the X‐ray beam.

## Supporting information

Supplementary Material

Supplementary Material

Supplementary Material

## References

[1] Sidhu M , Coley BD , Goske MJ , et al. Image gently, step lightly: increasing radiation dose awareness in pediatric interventional radiology. Pediatr Radiol. 2009;39(10):1135–38. doi:10.1007/s00247‐009‐1392‐5 19693493 10.1007/s00247-009-1392-5

[2] Strauss K , Butler PF , Goske MJ , Ritenour ER . Image gently: reducing radiation dose in pediatric computed tomography through collaboration. Med Phys. 2009;36(12):5719–20. 20095284 10.1118/1.3253462

[3] Strauss K , Goske MJ , Kaste SC , et al. Image gently: ten steps you can take to optimize image quality and lower CT dose for pediatric patients. AJR American J Roentgenol. 2010;194(4):868–73. doi:10.2214/AJR.09.4091 10.2214/AJR.09.4091 20308484

[4] Strauss KJ . Pediatric interventional radiography equipment: safety considerations. Pediatr Radiol. 2006;36 Suppl 2:126–35. doi:10.1007/s00247‐006‐0220‐4 10.1007/s00247-006-0220-4 PMC2663646 16862405

[5] Justino H . The ALARA concept in pediatric cardiac catheterization: techniques and tactics for managing radiation dose. Pediatr Radiol. 2006;36 Suppl 2:146–53. doi:10.1007/s00247‐006‐0194‐2 16862415 10.1007/s00247-006-0194-2 PMC2663648

[6] Belanger B and Boudry J . Management of pediatric radiation dose using GE fluoroscopic equipment. Pediatr Radiol. 2006;36 Suppl 2:204–11. doi:10.1007/s00247‐006‐0228‐9 16862403 10.1007/s00247-006-0228-9 PMC2663641

[7] Rogers DP , England F , Lozhkin K , Lowe MD , Lambiase PD , Chow AW . Improving safety in the electrophysiology laboratory using a simple radiation dose reduction strategy: a study of 1007 radiofrequency ablation procedures. Heart. 2011;97(5):366–70. doi:10.1136/hrt.2010.204222 hrt.2010.204222 [pii]. 21036800 10.1136/hrt.2010.204222

[8] Nicholson RA , Thornton A , Akpan M . Radiation dose reduction in paediatric fluoroscopy using added filtration. Br J Radiol. 1995;68(807):296–300. 7735770 10.1259/0007-1285-68-807-296

[9] Drury P and Robinson A . Fluoroscopy without the grid: a method of reducing the radiation dose. Br J Radiol. 1980;53(626):93–99. 7370517 10.1259/0007-1285-53-626-93

[10] Hernanz‐Schulman M , Goske MJ , Bercha IH , Strauss KJ . Pause and pulse: ten steps that help manage radiation dose during pediatric fluoroscopy. AJR Am J Roentgenol. 2011;197(2):475–81. doi:10.2214/AJR.10.6122 197/2/475 [pii] 21785097 10.2214/AJR.10.6122

[11] Sidhu M , Goske MJ , Connolly B , et al. Image gently, step lightly: promoting radiation safety in pediatric interventional radiology. AJR Am J Roentgenol. 2010;195(4):W299–W301. doi:10.2214/AJR.09.3938 195/4/W299 [pii] 20858793 10.2214/AJR.09.3938

[12] Sidhu M , Strauss K , Connolly B , et al. Radiation safety in pediatric interventional radiology. Tech Vasc Interv Radiol. 2010;13(3):158–66. doi:10.1053/j.tvir.2010.03.004 S1089‐2516(10)00013‐2 [pii] 20723830 10.1053/j.tvir.2010.03.004

[13] Ward VL , Strauss K , Barnewolt CE , et al. Pediatric radiation exposure and effective dose reduction during voiding cystourethrography. Radiology. 2008;249(3):1002–09. doi:10.1148/radiol.2492062066 18941159 10.1148/radiol.2492062066

[14] Gislason AJ , Davies AG , Cowen AR . Dose optimization in pediatric cardiac x‐ray imaging. Med Phys. 2010;37(10):5258–69. 21089760 10.1118/1.3488911

[15] McFadden SL , Hughes CM , Mooney RB , Winder RJ . An analysis of radiation dose reduction in paediatric interventional cardiology by altering frame rate and use of the anti‐scatter grid. J Radiological Prot. 2013;33(2):433–43. doi:10.1088/0952‐4746/33/2/433 10.1088/0952-4746/33/2/433 23612568

[16] King JM , Elbakri IA , Reed M . Antiscatter grid use in pediatric digital tomosynthesis imaging. J Appl Clin Med Phys. 2011;12(4):3641. doi:10.1120/jacmp.v12i4.3641 22089021 10.1120/jacmp.v12i4.3641 PMC5718745

[17] Lu ZF , Nickoloff EL , Ruzal‐Shapiro CB , So JC , Dutta AK . New automated fluoroscopic systems for pediatric applications. J Appl Clin Med Phys. 2005;6(4):88–105. 16421503 10.1120/jacmp.v6i4.2065 PMC5723454

[18] Partridge J , McGahan G , Causton S , et al. Radiation dose reduction without compromise of image quality in cardiac angiography and intervention with the use of a flat panel detector without an antiscatter grid. Heart. 2006;92(4):507–10. doi:hrt.2005.063909 [pii] 10.1136/hrt.2005.063909 16159965 10.1136/hrt.2005.063909 PMC1860862

[19] McFadden S , Hughes CM , D'Helft CI , et al. The establishment of local diagnostic reference levels for paediatric interventional cardiology. Radiography. 2013;19(4):295–301.

[20] Ubeda C , Vano E , Gonzalez L , Miranda P . Influence of the antiscatter grid on dose and image quality in pediatric interventional cardiology X‐ray systems. Catheter Cardiovasc Interv. 2013;82(1):51–57. doi:10.1002/ccd.24602 22899572 10.1002/ccd.24602

[21] Schueler BA , Julsrud PR , Gray JE , Stears JG , Wu KY . Radiation exposure and efficacy of exposure‐reduction techniques during cardiac catheterization in children. AJR Am J Roentgenol. 1994;162(1):173–77. doi:10.2214/ajr.162.1.8273659. 8273659 10.2214/ajr.162.1.8273659

[22] Kleinman PL , Strauss KJ , Zurakowski D , Buckley KS , Taylor GA . Patient size measured on CT images as a function of age at a tertiary care children's hospital. AJR Am J Roentgenol. 2010;194(6):1611–19. doi:10.2214/AJR.09.3771 194/6/1611 [pii] 20489103 10.2214/AJR.09.3771

[23] Soderman M , Holmin S , Andersson T , Palmgren C , Babic D , Hoornaert B . Image noise reduction algorithm for digital subtraction angiography: clinical results. Radiology. 2013;269(2):553–60. doi:10.1148/radiol.13121262 23737536 10.1148/radiol.13121262

[24] Racadio J , Strauss K , Abruzzo T , et al. Significant dose reduction for pediatric digital subtraction angiography without impairing image quality: a preclinical study in a piglet model. AJR Am J Roentgenol. 2014 Oct; 203(4):904–08. doi:10.2214/AJR.13.12170 25247959 10.2214/AJR.13.12170

[25] Pepe MS . The receiver operating characteristic curve. New York: Oxford University Press; 2003.

[26] Shrout PE and Fleiss JL . Intraclass correlations: uses in assessing rater reliability. Psychol Bull. 1979;86(2):420–28. 18839484 10.1037//0033-2909.86.2.420

