# Supplementary material for: Comparison of pediatric radiation dose and vessel visibility on angiographic systems using piglets as a surrogate: antiscatter grid removal vs. lower detector air kerma settings with a grid — a preclinical investigation
Source: J Appl Clin Med Phys. 2015 Sep 8;16(5):408–17. doi: 10.1120/jacmp.v16i5.5379 (PMC5690159; doi:10.1120/jacmp.v16i5.5379)
Supplement: Supplementary file 1 — Supplementary Material [file ACM2-16-408-s001.pdf]

American Association of Physicists in Medicine
One Physics Ellipse
College Park, MD 20740-3846
(301) 209-3350
Fax (301) 209-0862
http://www.aapm.org

Office of the Executive Director
Angela R. Keyser
Phone: 301-209-3385 Fax: 301-209-0862
E-mail: akeyser@aapm.org

DATE OF REQUEST: 09/23/14

FROM: Keith Strauss

EMAIL ADDRESS: Keith.Strauss@cchmc.org

1. Permission is granted to:

Keith Strauss

2. Permission is requested to use the following material:

K Strauss, R Nachabe and J Racadio, "WE-E-18A-10: Comparison of Patient Dose and Vessel Visibility Between Antiscatter Grid Removal and Lower Angiographic Radiation Dose Settings for Pediatric Imaging: A Preclinical Investigation." Med. Phys. 41(6) 512 (2014) Abstract.

3. For what purpose:

To publish my abstract in a manuscript with the Journal of Applied Clinical Medical Physics.

Permission is hereby granted: Angela R. Keyser 09/24/14

**Signature**	**Date**
