# Supplementary material for: Comparison of pediatric radiation dose and vessel visibility on angiographic systems using piglets as a surrogate: antiscatter grid removal vs. lower detector air kerma settings with a grid — a preclinical investigation
Source: J Appl Clin Med Phys. 2015 Sep 8;16(5):408–17. doi: 10.1120/jacmp.v16i5.5379 (PMC5690159; doi:10.1120/jacmp.v16i5.5379)
Supplement: Supplementary file 2 — Supplementary Material [file ACM2-16-408-s002.doc]

**Comparison of pediatric radiation dose and vessel visibility on angiographic systems: grid removal vs lower detector air kerma settings with a grid; a preclinical investigation**

**Keith J. Strauss, MSc**

Assistant Professor, University of Cincinnati School of Medicine

*Cincinnati Children’s Hospital Medical Center*

*MLC 5031, 3333 Burnet Avenue*

*Cincinnati, OH 45229-3026*

Phone: 513-803-1367

Cell: 617-908-7492

Fax: 513-636-8145

Email: [*keith.strauss@cchmc.org*](mailto:keith.strauss@cchmc.org)

Dose and IQ: grid vs non grid

**John M. Racadio, MD**

Professor, University of Cincinnati School of Medicine

**Todd A. Abruzzo, MD**

Associate Professor, University of Cincinnati School of Medicine

**Neil D. Johnson, MD**

Professor, University of Cincinnati School of Medicine

**Manish N. Patel, DO**

Assistant Professor, University of Cincinnati School of Medicine

*Cincinnati Children’s Hospital Medical Center*

*MLC 5031, 3333 Burnet Avenue*

*Cincinnati, OH 45229-3026*

Phone: 513-636-3385

Fax: 513-803-3015

Email: *john.racadio@cchmc.org*

**Kamlesh U. Kukreja, MD**

Section Chief, Interventional Radiology

Assistant Professor, Radiology, Baylor College of Medicine

*Texas Children’s Hospital*

*6701 Fannin Street*

*Suite 470*

*Houston, Texas 77030*

Phone*: 832-822-5324*

*Email:* [*drkamleshkukreja@yahoo.com*](mailto:drkamleshkukreja@yahoo.com)

**Mark.J.H. den. Hartog, MSc**

**Bart P.A. Hoornaert, MSc**

**Rami A. Nachabe, PhD**

*Philips Healthcare*

*Interventional X-ray Department*

*Building QY 2014A*

*Veenpluis 4-6*

*5680DA Best*

*The Netherlands*

Phone: 513-636-4599

Fax: 513-803-3015

Email: [*rami.nachabe@cchmc.org*](mailto:rami.nachabe@cchmc.org)

Abstract accepted and presented at 2014 Annual AAPM Meeting

Original Research

Keywords: Antiscatter grid, radiation dose, image quality, pediatric

[http://publishing](http://publishing/).aip.org/publishng/pacs/pacs-2010-regular-edition

Conflicts of Interest:

Cincinnati Children's Hospital Medical Center, Department of Radiology, Division of Interventional Radiology has a master research agreement with Philips Healthcare. Keith Strauss has had his travel expenses paid to perform research and development activities for Philips Healthcare. Keith Strauss has performed paid medical physics consulting services for Philips Healthcare upon their request. John Racadio has had his travel expenses paid for to Phillips sponsored symposiums. For the remaining authors none were declared. Mark den Hartog, Bart Hoornaert, and Rami Nachabe are employees of Philips Healthcare. However, the authors who are not Philips employees had full control of inclusion of any data and information that might present a conflict of interest for those authors who are Philips employees.
