# Supplementary material for: Comparison of pediatric radiation dose and vessel visibility on angiographic systems using piglets as a surrogate: antiscatter grid removal vs. lower detector air kerma settings with a grid — a preclinical investigation
Source: J Appl Clin Med Phys. 2015 Sep 8;16(5):408–17. doi: 10.1120/jacmp.v16i5.5379 (PMC5690159; doi:10.1120/jacmp.v16i5.5379)
Supplement: Supplementary file 3 — Supplementary Material [file ACM2-16-408-s003.docx]

ABSTRACT PRESENTED AT AAPM JULY 2014

Comparison of pediatric radiation dose and vessel visibility between antiscatter grid removal and lower angiographic radiation dose settings for pediatric imaging: a preclinical investigation

Purpose: To define an alternative to antiscatter grid (ASG) removal in angiographic systems which achieves similar patient dose reduction as ASG removal without degrading image quality during pediatric imaging.

Materials and Methods: This study was approved by the local institution animal care and use committee (IACUC). Six different digital subtraction angiography settings were evaluated that altered the mAs, (100, 70, 50, 35, 25, 17.5% of reference mAs) with and without ASG. Three pigs of 5, 15, and 20 kg (9, 15, and 17 cm abdominal thickness; smaller than a newborn, average 3 yr old, and average 10 year old human abdomen respectively) were imaged using the six dose settings with and without ASG. Image quality was defined as the order of vessel branch that is visible relative to the injected vessel. Five interventional radiologists evaluated all images. Image quality and patient dose were statistically compared using analysis of variance and receiver operating curve (ROC) analysis to define the preferred dose level and use of ASG for a minimum visibility of 2^nd^ or 3^rd^ order branches of vessel visibility.

Results: ASG grid removal reduces dose by 26% with reduced image quality. Only with the ASG present can 3^rd^ order branches be visualized; 100% mAs is required for 9 cm pig while 70% mAs is adequate for the larger pigs. 2^nd^ order branches can be visualized with ASG at 17.5% mAs for all three pig sizes. Without the ASG, 50%, 35% and 35% mAs is required for smallest to largest pig.

Conclusion: Removing ASG reduces patient dose and image quality. Image quality can be improved with the ASG present while further reducing patient dose if an optimized radiographic technique is used.
